# Other PHAC publications

**DOI:** 10.24095/hpcdp.45.4.07

**Published:** 2025-04

**Researchers from the Public Health Agency of Canada also contribute to work published in other journals and books. Look for the following articles published in 2024 and 2025:**

Betini RSD, Hussain M, Latus R, Chen A, Husak L, **Pelletier C, Shaver L**. Insights on the healthcare trajectories of people living with dementia. Healthc Q. 2024;27(3):11-4. https://doi.org/10.12927/hcq.2024.27493

Catherine NLA, Macmillan H, Jack S, Zheng Y, Xie H, Boyle M, […] **Tonmyr L**, et al. Effects of nurse-home visiting on intimate partner violence and maternal income, mental health and self-efficacy by 24 months postpartum: a randomised controlled trial (British Columbia Healthy Connections Project). BMJ Open. 2025;15(1):e083147. https://doi.org/10.1136/bmjopen-2023-083147

Emeljanovas A, Mieziene B, Venckunas T, **Lang JJ**, Tomkinson GR. Trends in physical fitness among Lithuanian adolescents aged 11-17 years between 1992 and 2022. J Epidemiol Community Health. 2024. https://doi.org/10.1136/jech-2024-223072

Hundal S, **Cappelli J**, Croitoru D, Drucker AM, Ingram JR, Goldberg SR, et al. Cost-utility analysis of clinic-based deroofing versus local excision for hidradenitis suppurativa. J Am Acad Dermatol. 2024. https://doi.org/10.1016/j.jaad.2024.11.057

Mok N, **Knox NC**, Zhu F, Arnold DL, Bar-Or A, Bernstein CN, **Bonner C**, […] **Graham M**, […] **Van Domselaar G**, et al. The fungal gut microbiota in pediatric-onset multiple sclerosis. Front Microbiol. 2024;15:1258978. https://doi.org/10.3389/fmicb.2024.1258978

**Pollock NJ**, Yantha C, **Tonmyr L**, Jewers-Dailley K, Morton Ninomiya ME. Child welfare worker perspectives on documentation and case recording practices in Canada: a mixed-methods study protocol. PLoS ONE. 2025;20(1):e0316238. https://doi.org/10.1371/journal .pone.0316238

**Shi Y, Macrae K, de Groh M, Thompson W**, Stockwell T. Mortality and hospitalizations fully attributable to alcohol use before versus during the COVID-19 pandemic in Canada. CMAJ. 2025;197(4):E87-95. https://doi.org/10.1503/cmaj.241146

